# Photocurable Methacrylate Derivatives of Polylactide: A Two-Stage Synthesis in Supercritical Carbon Dioxide and 3D Laser Structuring

**DOI:** 10.3390/polym12112525

**Published:** 2020-10-29

**Authors:** Vladislav S. Kaplin, Nikolay N. Glagolev, Valentina T. Shashkova, Irina A. Matveeva, Ilya V. Shershnev, Tatyana S. Zarkhina, Nikita V. Minaev, Nadezhda A. Aksenova, Boris S. Shavkuta, Evgeny A. Bezrukov, Aleksandr S. Kopylov, Daria S. Kuznetsova, Anastasiia I. Shpichka, Peter S. Timashev, Anna B. Solovieva

**Affiliations:** 1 Semenov Federal Research Center of Chemical Physics, 4 Kosygin St., 119991 Moscow, Russia; nikgl@mail.ru (N.N.G.); persik-oo@mail.ru (V.T.S.); n.buev@mail.ru (I.A.M.); zarkhina@mail.ru (T.S.Z.); naksenova@mail.ru (N.A.A.); kopylov.a.s.86@gmail.com (A.S.K.); shershnev.ilya@gmail.com (I.V.S.); timashev.peter@gmail.com (P.S.T.); ann.solovieva@gmail.com (A.B.S.); 2 Research center “Crystallography and Photonics”, Institute of Photonic Technologies, 2 Pionerskaya St., Troitsk, 108840 Moscow, Russia; minaevn@gmail.com (N.V.M.); b.shavkuta@gmail.com (B.S.S.); 3 Institute for Regenerative Medicine, Sechenov University, 8 Trubetskaya St., 119991 Moscow, Russia; ana-shpichka@yandex.ru; 4 Institute for Urology and Reproductive Health, Sechenov University, 8 Trubetskaya St., 119991 Moscow, Russia; eabezrukov@rambler.ru; 5 Institute of Fine Chemical Technologies, Russian Technological University, 78 Vernandsky Avenue, 119454 Moscow, Russia; 6 Institute of Experimental Oncology and Biomedical Technologies, Privolzhsky Research Medical University, Minin and Pozharsky Sq. 10/1, 603950 Nizhny Novgorod, Russia; daria.s.kuznetsova@gmail.com; 7 Chemistry Department, Lomonosov Moscow State University, 1-3 Leninskiye Gory, 119991 Moscow, Russia

**Keywords:** polylactide, supercritical carbon dioxide, photopolymerization, two-photon polymerization, tissue engineering

## Abstract

A two-stage polylactide modification was performed in the supercritical carbon dioxide medium using the urethane formation reaction. The modification resulted in the synthesis of polymerizable methacrylate derivatives of polylactide for application in the spatial 3D structuring by laser stereolithography. The use of the supercritical carbon dioxide medium allowed us to obtain for the first time polymerizable oligomer-polymer systems in the form of dry powders convenient for further application in the preparation of polymer compositions for photocuring. The photocuring of the modified polymers was performed by laser stereolithography and two-photon crosslinking. Using nanoindentation, we found that Young’s modulus of the cured compositions corresponded to the standard characteristics of implants applied in regenerative medicine. As shown by thermogravimetric analysis, the degree of crosslinking and, hence, the local stiffness of scaffolds were determined by the amount of the crosslinking agent and the photocuring regime. No cytotoxicity was observed for the structures.

## 1. Introduction

**Figure 1:**
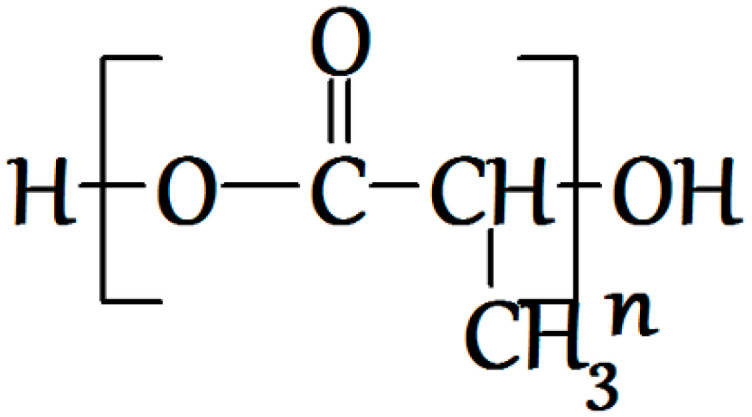
The structural formula of poly(lactic) acid (PLA).

Polymeric biocompatible materials have been in special demand in the new field of medical materials science, tissue engineering, associated with reconstructive surgery and the development of artificial organs [1]. Indeed, to design a new generation of implants applied in the replacement of lost or damaged tissues and organs, one needs materials with variable physicomechanical characteristics, which cause a minimum tissue reaction [2,3,4]. One type of such material is polyesters of aliphatic hydrocarboxylic acids, first of all, polylactide acid (polylactide, PLA) (Figure 1). Due to the specifics of its production and destruction, PLA occupies one of the basic places in the series of biocompatible and biodegradable polymers used in regenerative medicine and plastic surgery for reconstruction of bone and cartilage defects [5,6]. Besides, PLA is a thermoplastic polymer and may be processed by extrusion, molding, and blow molding. However, when using PLA as an initial polymer matrix for the implant design, it becomes necessary to alter a number of its characteristics, first of all, its fragility, low porosity, low thermal stability, and high hydrophobicity, leading to low cell adhesion to the material’s surface [7,8].

To improve cell adhesion and increase hydrophilicity and bactericidal properties of polylactides, a laser or plasma treatment of the polymer materials’ surface is used [9,10], with the addition of crosslinking agents carrying reactive groups—carboxyl, hydroxyl, amino groups, or their combination [11,12,13], as well as grafting of polymers with antibacterial properties [14].

The bulk properties of PLA, in particular, hydrophobicity, may be varied by synthesizing copolymers of polylactide with carbonates, lactones, glycolides, glycols, urethanes, etc. [15], or by graft-copolymerization, e.g., introduction of monomeric lactide in a hydrophilic polyethyleneglycol macromolecule [16].

Chemical modification of the PLA terminal groups may appear a promising approach to enhancing PLA hydrophilicity and affinity to cells with the simultaneous decrease of the polymer fragility. Copolymers of lactic acid with hydrophilic monomers are usually synthesized for this purpose. In particular, PLA copolymers with starch are obtained in toluene at 150 °C, followed by azeotropic dehydration [17], or by extrusion at 180 °C [18]. PLA copolymers with polyethyleneoxide are synthesized in toluene in a nitrogen atmosphere at 85 °C [19]. One of the most promising approaches to the structuring of polylactide (as well as other polymers containing hydroxyl groups, e.g., chitosan [20]), in which modification of the PLA terminal groups is applied, is the utilization of various techniques of thermal and photocuring, including laser stereolithography [21]. To accomplish this, new functional groups containing unsaturated bonds are added to PLA hydroxyl groups via esterification [22]. As the modifying agents, anhydride [23,24] or chloroanhydride of methacrylic acid [25] are most frequently used. Such reactions proceed in the presence of pyridine [26] or trimethylamine [27,28] in the liquid medium: in toluene [29], dimethylformamide [26], or haloalkanes [30]. One may also graft the double bonds to polyesters of hydroxyacids using a more advanced approach of urethane formation, with the use of diisocyanates and hydroxyl-containing modifying agents [31,32]. However, this method is only scantily described in the literature, though such reactions do not require toxic substances, such as anhydrides and chloroanhydrides of acids, tertiary amines, or pyridine [33]. This fact is probably related to the difficulties arising due to a higher sensitivity of urethane formation to the presence of even insignificant moisture, with the need to dry the reactants and the solvent and to conduct the reaction in an inert atmosphere.

Earlier, we performed a modification of polylactide with the introduction of polymerizable acrylate groups into the macromolecule via the reactions of esterification and urethane formation of the terminal carboxyl and hydroxyl groups in a toluene or methylene chloride solution, respectively, which allowed obtaining 3D crosslinked structures by photopolymerization [29,34]. It should also be noted that all the above-mentioned ways of synthesis in the liquid phase involve the stage of product separation: reprecipitation, centrifugation, washing, and long drying. This leads not only to product losses but also to obtaining a resin-like mass as a result, inconvenient for further application since it is almost impossible to remove the solvent completely in common conditions. As reported in [22,35], a partial self-crosslinking takes place in such a case, resulting in the shelf life of the product not exceeding 1–2 months. We showed earlier that the esterification reaction in a solution had a degree of conversion limited by 65% [29]. When using urethane formation for PLA modification in a solution, we managed to raise the degree of conversion to 92%; however, the product state as a viscous mass did not allow its laser structuring application due to proceeding self-crosslinking [34].

Carbon dioxide is most frequently used as a supercritical state solvent for conducting various chemical reactions, that is, primarily related to its convenient critical parameters (31.2 °C, 7.3 MPa) and affinity to both polar and nonpolar polymer matrices. Besides, supercritical carbon dioxide (scCO_2_) is capable of dissolving organic molecules of different polarities and disappearing from the system after the process completion that facilitates the separation and purification of products [36,37]. Earlier, using the scCO_2_ medium, the bulk properties of ultra-high-molecular-weight polyethylene (UHMWPE) were modified by the synthesis of crosslinked methacrylate systems inside the UHMWPE matrix, which formed interpenetrating network structures with interesting mechanical properties [38,39]. In particular, it was noted that mixing UHMWPE with polymethylmethacrylate-co-poly(ethyleneglycol)dimethacrylate resulted in the formation of an extremely strong crosslinked material, which did not exhibit the usual thermal deformation behavior observed for UHMWPE.

In the field of creation of biodegradable implants, supercritical CO_2_ has already found application as a pore-forming agent [40,41] or for sterilization [42]. However, the use of scCO_2_ as a medium for chemical modification of polylactide and its derivatives has almost no mentions, despite its advantages of inertness and the absence of moisture, rapid removal from the reaction mixture, and absence of toxicity.

In this study, supercritical CO_2_ was used as a solvent for urethane formation for the first time. The study objective was an elaboration of an approach for the synthesis of methacrylate PLA derivatives via the urethane formation reaction (from the interaction of polylactide hydroxyl groups with diisocyanates) in the medium of supercritical carbon dioxide. The reaction was conducted in two stages with the separation of the intermediate diisocyanate PLA derivative, followed by its interaction with ethyleneglycol monomethacrylate, leading to the formation of PLA containing polymerizable methacrylic groups. Using laser stereolithography and two-photon crosslinking, we prepared 3D crosslinked structures based on the synthesized PLA methacrylate and tested them for cytotoxicity.

## 2. Materials and Methods

### 2.1. PLA Modification Process

**Figure 2:**
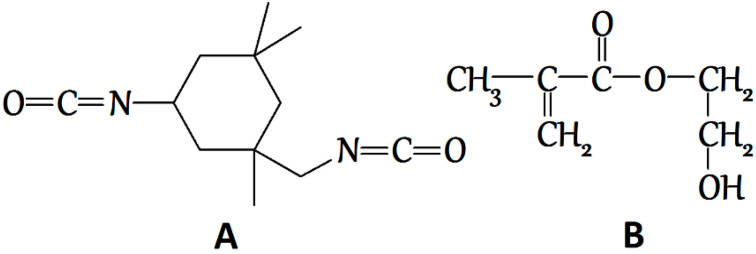
Structural formulas of the reactants used: (**A**)—3-isocyanatomethyl-3,5,5-trimethylcyclohexyl isocyanate (ITI), (**B**)—ethyleneglycol monomethacrylate (EGM).

PLA methacrylation via the urethane formation reaction was performed with the use of 3-isocyanatomethyl-3,5,5-trimethylcyclohexyl isocyanate (ITI, 98%, a mixture of isomers, Aldrich, St. Louis, MO, USA) (Figure 2A) and ethyleneglycol monomethacrylate (EGM, Aldrich, St. Louis, MO, USA) (Figure 2B). Polylactide with the average molecular weight of 5 × 10^3^ Da (Aldrich, St. Louis, MO, USA) was used for the modification. All the components were used without additional purification. Dry carbon dioxide, with the volume content of water vapor not exceeding 0.001%, according to the quality certificate, was utilized. The solubility of the initial reactants in the scCO_2_ medium was tested as follows: each of the reactants was placed into a steel reactor with a volume of 3 mL and quartz windows, in a concentration corresponding to its concentration in the reaction. In the reactor, we reproduced the scCO_2_ medium at the temperature and pressure corresponding to the reaction conditions. Then, we registered a UV-Vis absorption spectrum using a Cary 50 spectrophotometer (Agilent Technologies, Santa Clara, CA, USA) and compared the acquired spectrum with the absorption spectrum of the same substance in chloroform.

**Figure 3:**
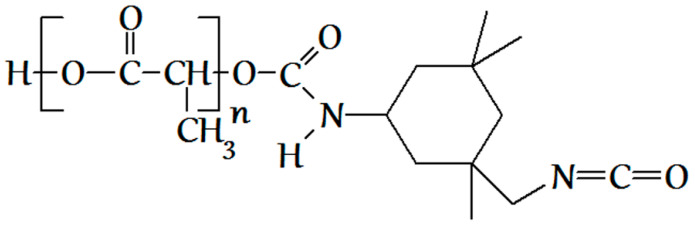
The structural formula of the isocyanate PLA derivative, obtained in the first stage.

**Figure 4:**
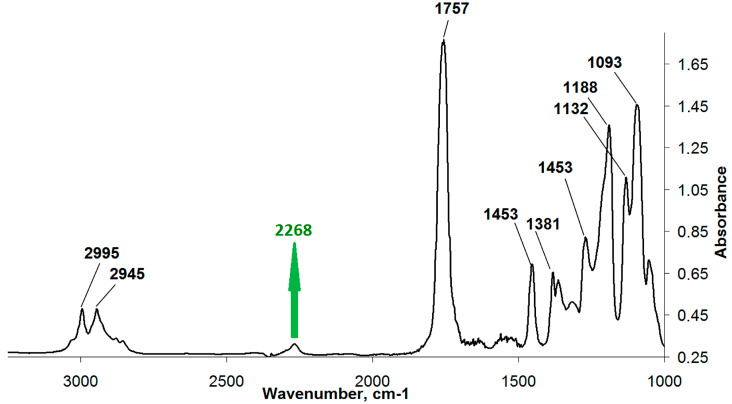
IR spectrum of the reaction mixture obtained at the first stage, after its purification by reprecipitation.

**Figure 5:**
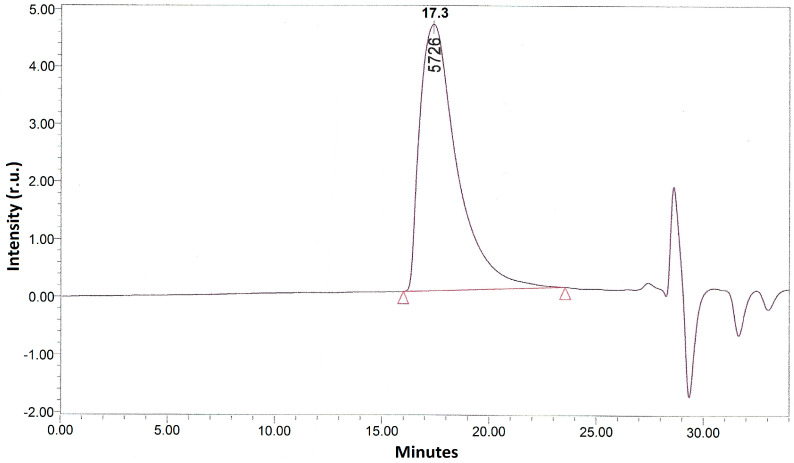
SEC (size-exclusion chromatography) elution profile of the reaction mixture obtained at the first stage, after its purification by reprecipitation. The red line and triangles indicate the signal boundaries of the main reaction product.

As mentioned above, the synthesis of the methacrylate PLA derivative in scCO_2_ was conducted in two stages. At the first stage, PLA isocyanate (Figure 3) was obtained by the interaction of PLA hydroxyl groups with ITI isocyanate groups as follows: 0.001 mol of PLA and 0.0011, 0.002, and 0.003 mol of ITI (with the excess of 10%, 100%, and 200%, respectively, with respect to PLA), as well as 0.15 mL of the catalyst—dibutyltin dilaurate (Aldrich, St. Louis, MO, USA)—was placed into a steel reactor with the volume of 78 cm^3^ containing a magnetic anchor. After that, the reactor was filled with gaseous CO_2_ at a pressure of about 6 MPa at 25 °C and heated to 40 °C; in doing so, the pressure increased to 9 MPa, and carbon dioxide underwent a transition first to the liquid and then to the supercritical state (the whole process takes about 8 min). The reaction was conducted for either 10 or 20 h, and then CO_2_ was removed. The completion of the first stage was controlled using IR spectroscopy and size-exclusion chromatography (SEC) via the appearance and growth of the isocyanate band (2268 cm^−1^) until its intensity became constant (Figure 4) in the IR spectrum of the reaction mixture purified by reprecipitation, which, according to the SEC data (Figure 5), was free from the unreacted three-fold excess of ITI (which is usually present at 25.7 min at a chromatogram of the unpurified isocyanate polylactide derivative (Figure S1)).

**Figure 6:**
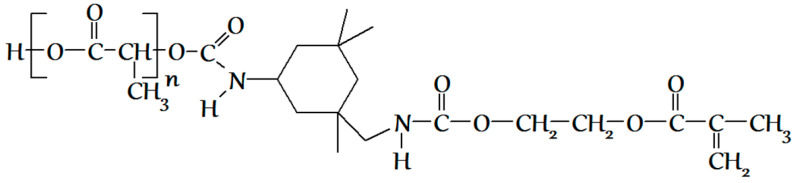
The structural formula of the methacrylate PLA derivative, obtained at the second stage.

**Figure 7:**
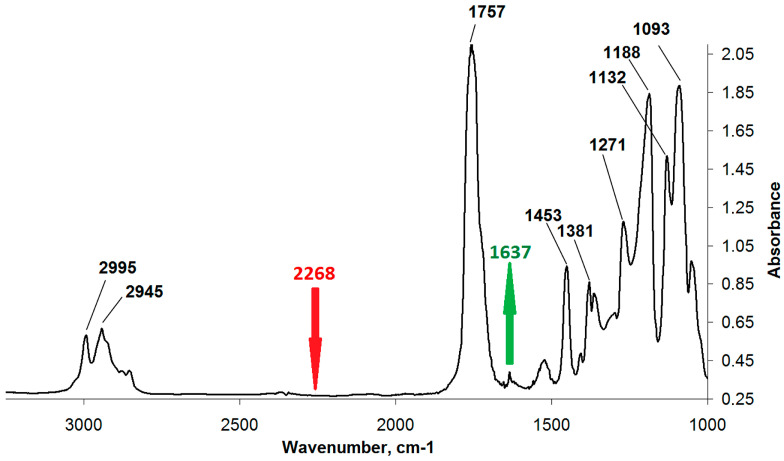
IR spectrum of the reaction mixture obtained at the second stage, after its purification by reprecipitation.

**Figure 8:**
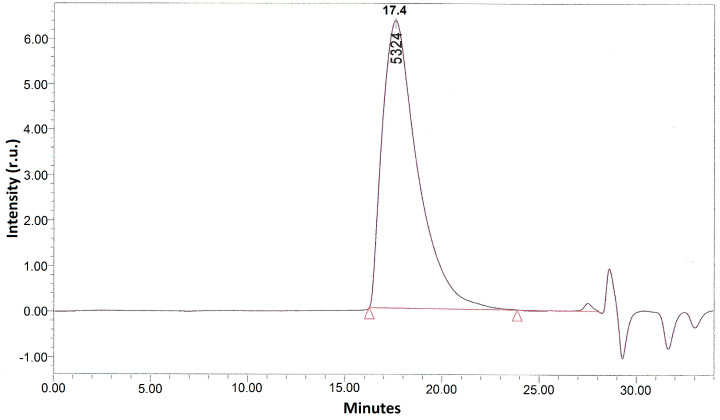
SEC elution profile of the reaction mixture obtained at the second stage, after its purification by reprecipitation. The red line and triangles indicate the signal boundaries of the main reaction product.

In the second stage, the methacrylate PLA derivative (Figure 6) was obtained by the interaction of isocyanate groups of PLA derivative obtained at the first stage with the EGM hydroxyl groups. EGM in the amount of 0.0011, 0.003, or 0.005 mol, corresponding to the excess of 10%, 200%, or 400%, respectively, to PLA, and 0.15 mL of dibutyltin dilaurate as a catalyst were loaded into the reactor containing the reaction mixture obtained at the first stage (PLA isocyanate and unreacted ITI). The reaction was conducted in the scCO_2_ medium in the same conditions, like those at the first stage, for 10 h. The completion of the methacrylate PLA derivative formation was also controlled by SEC and IR spectroscopy via the disappearance of the isocyanate band (2268 cm^−1^) and appearance and growth of the double bond vibration band (1637 cm^−1^) (Figure 7) in the IR spectrum of the reaction mixture purified by reprecipitation, which, according to the SEC data (Figure 8), was free from the unreacted five-fold excess of EGM (with only one main product present). The reaction mixture after the CO_2_ removal represented a dry powder, which was dissolved in chloroform and precipitated in a ten-fold hexane excess for purification, separation, and analysis of the final product. The degree of polylactide modification was measured as follows: a charge of the final product containing both modified and unreacted PLA (m_a_) was dissolved in dichloromethane, then a photoinitiator and a curing agent (oligourethanedimethacrylate, OUDM) were added, and photocuring was conducted using a scattered light of a mercury lamp according to the procedure described in [34]. Then, the prepared crosslinked composition was extracted with tetrahydrofuran of a volume V_e_ for 24 h. As a result, an extract of a volume V_e_ was prepared, containing the entire unmodified PLA, which did not participate in the photocuring reaction. Using a calibration plot of the PLA peak area vs. PLA concentration, we calculated the unmodified PLA concentration in the extract (C). Then, the overall weight of the unmodified PLA extracted from the photocured charge was calculated (C*V_e_). Based on the obtained values, the degree of PLA modification was calculated according to Formula (1).

(m_a_ − m_b_)/m_a_ × 100% (1)

The analysis of the products was conducted by SEC with a Waters chromatograph (Milford, MA, USA) (Breeze system, Ultrasilicagel 100 Å, 500 Å, and 1000 Å standard columns, 1 mL/min, detectors—a refractometer and a UV-module with a variable wavelength) and FTIR spectroscopy (Varian 800, Agilent Technologies, Santa Clara, CA, USA).

To confirm the formation of the isocyanate and methacrylate PLA derivatives, we also conducted an NMR study. ^1^H- and ^13^C-NMR spectra of the half-product and product were acquired with a Bruker Avance 600 spectrometer (Billerica, MA, USA). To purify the substances from the unreacted reactants and side-products, they were dissolved in dichloromethane with the following reprecipitation in a ten-fold excess of hexane. The spectra were registered in a CDCl_3_ solution at the temperature of 25 °C and the frequency of 600 MHz (^1^H-NMR) and 150 MHz (^13^C-NMR).

PLA isocyanate derivative: ^1^H-NMR (CDCl_3_, 600 MHz) δ, ppm: 0.88 (t, 9H, CH3_c,d_), 0.93–1.11 (15H, CH3_c,d_, CH2_e_), 1.26–1.30 (m, 6H, CH2_e_), 1.47–1.66 (m, 3H, CH3_b_), 2.37 (m, 1H, CH_h_), 3.05 (s, 2H, CH2_f_), 4.73 (m, 1H, NH_g_), 5.13–5.24 (m, 1H, CH_a_).

PLA methacrylate derivative: ^1^H-NMR (CDCl_3_, 600 MHz) δ, ppm: 0.88 (t, 9H, CH3_c,d_), 0.93–1.11 (15H, CH3_c,d_, CH2_e_), 1.25–1.31 (m, 6H, CH2_e_), 1.47–1.60 (m, 3H, CH3_b_), 1.95 (s, 3H, CH3_n_), 2.32 (t, 1H, CH_h_), 2.92 (m, 2H, CH2_f_), 4.32 (m, 4H, CH2_j,k_), 4.63 (d, 1H, NH_g_), 4.86 (t, 1H, NH_i_), 5.13–5.24 (m, 1H, CH_a_), 5.59 (s, 1H, CH2_m,l_), 6.14 (s, 1H, CH2_m,l_).

### 2.2. Preparation of Crosslinked Structures

The crosslinked samples represented either films with a diameter of 8–10 mm (and the film thickness of 1 mm) or scaffolds. To obtain films, a DRT-1000 mercury lamp (with the intensity of incident unfiltered light I ≈ 0.03 W/cm^2^, exposure of 300 s) [34] or a powerful (50 W) light emitting diode (LED) matrix (Epileds Technologies Inc., Tainan, Taiwan, λ = 365 nm, I = 5 W/cm^2^, exposure of 200 s, out-of-focus light) were used. The photoactive composition was a 12% PLA methacrylate solution in methylene chloride containing a crosslinking agent—oligourethanedimethacrylate (OUDM) (2.3–20 wt. %)—and a photoinitiator (Michler’s ketone (Aldrich, St. Louis, MO, USA), PI, 5 wt. % with respect to the weight of the modified PLA). The composition (in the form of a viscous liquid with a volume of about 0.3 mL) was placed onto Teflon support (3 cm × 3 cm). It appeared possible to use the unprecipitated reaction mixture with the addition of the deficient amount of the crosslinker and photoinitiator as a photoactive composition.

Scaffolds were prepared by laser stereolithography [43] and two-photon crosslinking [44,45]. Samples with the volume of 60 µL were fabricated by layer-by-layer deposition of 15 µL of the photoactive composition on a coverslip. The air-dried composition contained approximately 10 wt. % of the residual solvent. The crosslinked samples after photocuring were kept in tetrahydrofuran for a day to remove the unreacted polymer, as well as unreacted reactants and side products of the reaction, then dried on air.

### 2.3. Studies of Mechanical Characteristics

Local Young’s moduli of crosslinked samples were measured with a Piuma Nanoindenter (Optics11, Amsterdam, The Netherlands) using a cantilever with a spring constant of 52.4 N/m and a tip radius of 30.0 µm. The measurements were conducted on air. The area of Young’s modulus maps was 300 × 300 µm^2^ with an increment of 30 µm by X and Y axes. Based on the measurement results, the effective Young’s modulus (E) of a sample was calculated (mean ± standard deviation (SD)).

### 2.4. Differential Thermal Analysis

For the differential thermal analysis (DTA), a synchronous STA 449 F3 thermal analyzer (NETZCH, Selb, Deutschland) was used. The destruction process was conducted on air at the gas flow rate of 30 mL/min and a linear heating rate of 10 °C/min, and the samples’ charges were 2–5 mg. The weight losses were registered with the accuracy up to 10^−3^ mg, and the relative error of temperature measurement was ±1.5 °C. The weight loss (thermogravimetric analysis, TGA) and the rate of the weight loss (W) dependencies on the temperature were studied. As quantitative characteristics of the proceeding thermooxidative destruction of PLA methacrylate derivatives, obtained by different photocuring techniques, we used the weight loss (Δm, %), maximum destruction rate (W_Max_, %/min), and coke residue (coke, wt. %).

### 2.5. Cell Culture

For cytotoxicity assays, we used a 3T3 mouse fibroblast cell line (Biobank, Sechenov University, Moscow, Russia) Cells were cultured, as described elsewhere [46,47]: under standard conditions (37 °C, 5% CO_2_) in DMEM/F12 (1:1) basal medium (Gibco) supplemented with a 10% FBS (HyClone) and 1% penicillin-streptomycin (Gibco). Every 2–3 days, we changed the medium. Cells were passaged when they reached 80% confluency.

### 2.6. Cytotoxicity Assessment

The possible cytotoxicity of the synthesized material was assessed using Live/Dead (Sigma Aldrich, St. Louis, MO, USA) staining and AlamarBlue (Invitrogen, Carlsbad, CA, USA) and MTT (Sigma Aldrich, St. Louis, MO, USA) assays. To reveal live and dead cells, we seeded scaffolds with the previously described design [48,49,50] with 1 × 10^5^ cells (per scaffold) and stained them for 72 h with calcein-AM (live, green) and propidium iodide (dead, red) (Sigma Aldrich, St. Louis, MO, USA); cell nuclei were additionally stained with Hoechst 33342 (blue). For AlamarBlue and MTT assays, we inoculated 5 × 10^3^ cells to each well in a 96-well plate and added a matrix extract or sodium dodecyl sulfate (positive control, in seven dilutions) in 24 h. The matrix extract was prepared by dipping its part with the surface area of 6 cm^2^ into 1 mL DMEM/F12 supplemented with 5% FBS and 1% penicillin-streptomycin and placing for 24 h in a thermostat (ISO 10993-12:2012). The AlamarBlue and MTT assays were performed in accordance with the manufacturer’s instructions.

## 3. Results and Discussion

### 3.1. A Two-Stage Synthesis of Polylactide Modified with Functional Groups in the scCO_2_ Medium

It is known that one may introduce unsaturated methacrylic groups to macromolecules containing reactive hydroxyl groups (polyols, glycols, and hydroxycarboxylic acids) via the reaction of urethane formation performed in one or two stages [51]. The single-stage synthesis is the simplest way of modification; however, the lower reactivity of PLA terminal hydroxyl groups with respect to the EGM hydroxyl group leads to a competitive reaction of the interaction between EGM and ITI. Taking into account this circumstance, to increase the degree of PLA modification, we performed methacrylation in the supercritical carbon dioxide medium in two stages, obtaining the isocyanate PLA derivative at the first stage. All the initial reactants, including polylactide, as well as intermediate and final products, are shown to be soluble in the scCO_2_ medium; thus, the reaction in scCO_2_ proceeded in the homogeneous conditions.

To boost the degree of modification of the methacrylate polylactide derivative, we used a molar excess of ITI (by 1.1, 2.0, and 3.0 times at the first stage) and EGM (by 1.1, 3.0, and 5.0 times at the second process stage) with respect to PLA. The reaction time for the PLA isocyanate derivative synthesis is also increased from 10 to 20 h.

**Figure 9:**
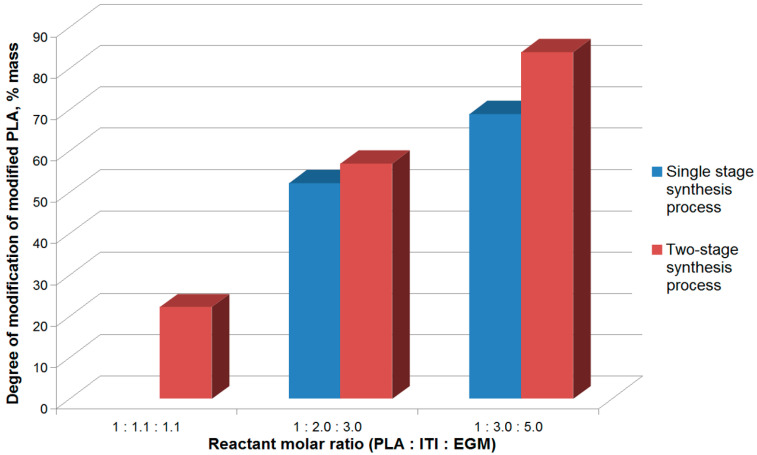
Average methacrylated PLA degree of modification in single-stage and two-stage reactions of PLA modification in the scCO_2_ (supercritical carbon dioxide) medium (40 °C, 9 MPa) at different ratios of initial components, calculated by the formula (1).

It should be noted that usually when obtaining isocyanate derivatives of polymers containing hydroxyl groups, a 10% excess of the isocyanate and methacrylating agent is used [34]. However, as follows from Figure 9, at such ratios of components, the degree of modification of methacrylated PLA is only 20% due to the low reactivity of PLA terminal hydroxyl groups. In the case of the increased ITI and, hence, EGM content in the system, the degree of modification of methacrylated PLA grows (Figure 9). At the same time, the excess of the methacrylating agent (EGM) with respect to the isocyanate component is related to the necessity of binding toxic isocyanate groups not having reacted with PLA. It should be noted that the increase of the ITI and EGM content in the system above the mentioned ratios does not result in the increase of the methacrylated PLA degree of modification, probably, due to a dramatic viscosity rise.

The reaction mixture obtained at the first stage of the process and consisting of PLA isocyanate and unreacted ITI is introduced into the reaction with EGM, which results in the formation of the PLA methacrylate derivative (Figure 9). Thus, the use of the large excess of ITI and EGM and the two-stage design of the reaction allows achieving an 82% degree of PLA modification in the scCO_2_ medium. The obtained degree of conversion is the mean value after the no less than ten-fold experiment reproduction and is as high as those in alternative reactions of PLA methacrylation [26,27,28]. However, in the scheme, we suggested there is no stage of solvent removal, which is unavoidable when the reaction is conducted in the liquid phase. After the CO_2_ release, the final reaction mixture represents a stable dry powder, which may be stored for no less than a year without spontaneous self-crosslinking. Secondly, as will be shown further, the reaction mixture does not require additional purification after the second stage and may be used immediately as a base for a photoactive composition.

**Figure 10:**
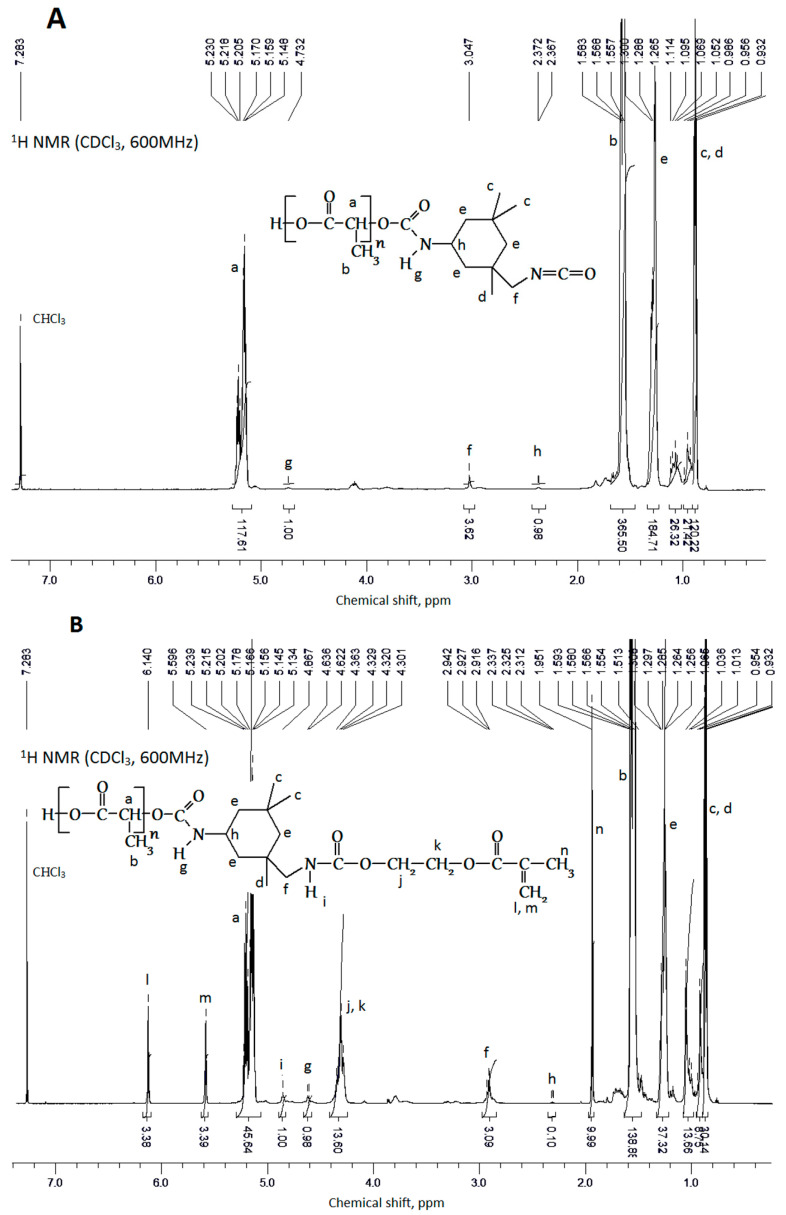

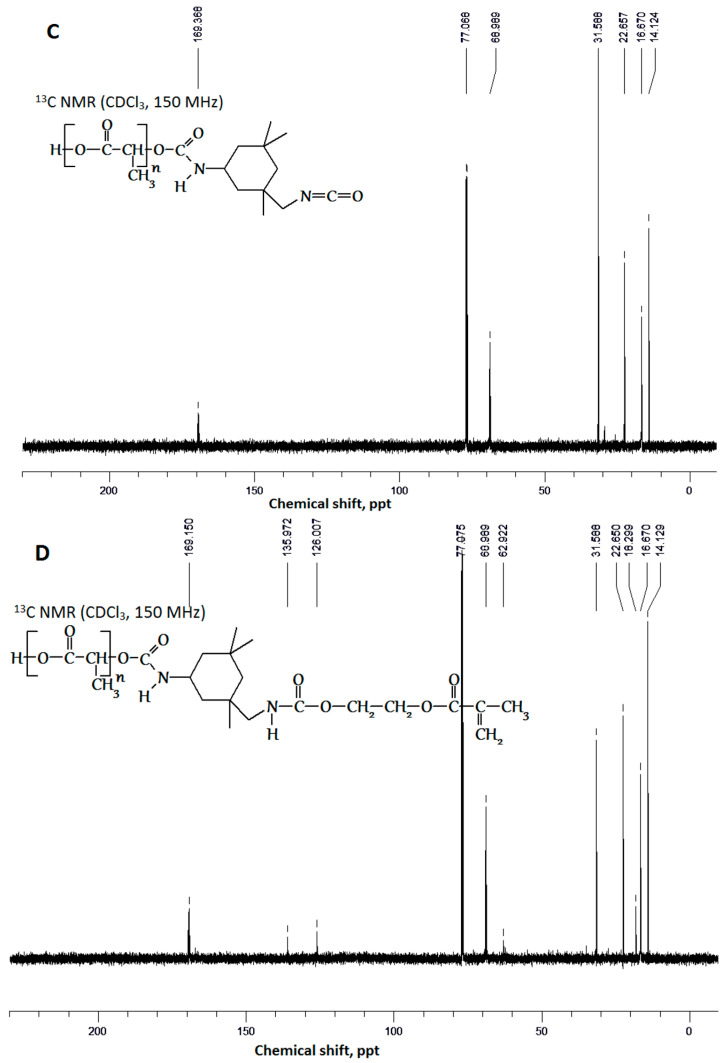
(**A**) The ^1^H-NMR spectrum of the PLA isocyanate derivative, obtained at the first stage. (**B**) The ^1^H-NMR spectrum of the PLA methacrylate derivative, obtained at the second stage. (**C**) The ^13^C-NMR spectrum of the PLA isocyanate derivative, obtained at the first stage. (**D**) The ^13^C-NMR spectrum of the PLA methacrylate derivative, obtained at the second stage.

The addition of the isocyanate and then methacrylate fragments to PLA molecules is confirmed by ^1^H- and ^13^C-NMR spectra. The ^1^H-NMR spectra of the initial reactants (PLA, ITI, EGM) is shown in the Supplementary Materials (Figure S2c). The analysis shows that the ^1^H-NMR spectrum of a sample of the isocyanate PLA derivative (Figure 10A) contains only signals from the polylactide chain (a, b) and ITI (c–h). The reaction is confirmed by the appearance of signals of the “g” urethane group protons near 4.73 ppm and by the shift of signals of the “h” protons of the tertiary carbon atom to a stronger field (Figure 10A). With the formation of the methacrylate derivative, new signals appear in addition to the earlier observed ones, which belong to the monomethacrylate ester of ethyleneglycol (i–n) (Figure 10B). A signal appears, which is assigned to the “i” proton of the second urethane fragment, and a shift of the “f” proton signals towards a stronger field also occurs. The addition of monoethyleneglycol ester to the polymer chain of polylactide is also proven by the ^13^C-NMR spectroscopy data (Figure 10C,D). The appearance of signals in the region of 120–140 ppm in the spectrum of the methacrylate derivative indicates the methacrylate residue having embedded into the polymer.

It should be noted that the presence of the ITI and EGM excess in the reaction mixture leads to side reactions proceeding with the formation of 2–9% of oligourethanedimethacrylate (OUDM), a product of the ITI interaction with EGM in the ratio of 1:2 [34]. However, the so-formed oligourethanedimethacrylates are shown to be able to play the role of crosslinking agents in the photopolymerization of modified PLA. Other side products, as well as unreacted reactants, appear not to hinder the photocuring reactions and lower the quality of the prepared scaffolds. All these components are readily removed from the prepared crosslinked structure during its washing. Thus, the reaction mixture may be used as a basis for a photoactive composition as is, without any purification. The following addition of more OUDM and PI is only needed for the preparation of 3D crosslinked compositions with optimal physicochemical characteristics. All these findings make the obtained dry reaction mixture very convenient for fast preparation of a photoactive composition and creation of scaffolds by laser stereolithography.

### 3.2. The Effect of the Crosslinking Agent (OUDM) Concentration on the Structure of Photocured Compositions

**Figure 11:**
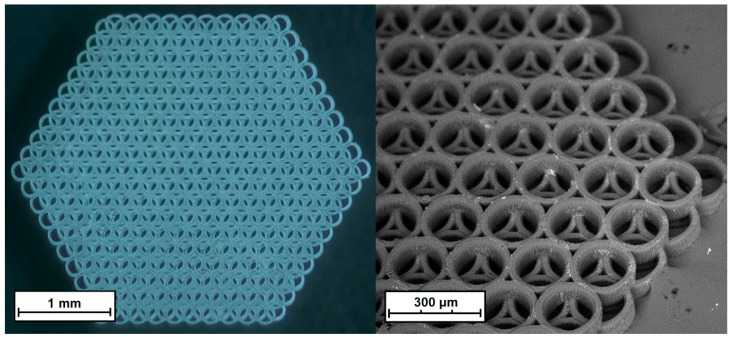
A 3D crosslinked structure (scaffold), prepared as a result of two-photon polymerization of a composition consisting of modified methacrylate-containing PLA, 15 wt. % of OUDM (oligourethanedimethacrylate), and 5 wt. % of the photoinitiator.

As mentioned above, oligourethanedimethacrylate, formed as a side product at the second stage of synthesis from the ITI (remaining in the reaction mixture after the first stage of the process) interaction with EGM, plays a role of a crosslinking agent in the photopolymerization reaction of methacrylated PLA. At the same time, the OUDM content in the photoactive composition appears to influence the stability of the obtained 3D crosslinked structures. Indeed, at the OUDM content of less than 10 wt. %, no formation of a stable crosslinked structure (insoluble in organic solvents) takes place. On the other hand, the addition of more than 17 wt. % of the crosslinking agent leads to an increase of fragility and the sample’s destruction. The most stable 3D-crosslinked structures are achieved at the 15 wt. % OUDM content in the composition. Photoactive compositions with such content are used in further tests. In particular, based on such compositions, crosslinked structures with a size of up to 3 mm are prepared, which are used in the cytotoxicity testing after their storage in tetrahydrofuran and water (Figure 11).

As we already mentioned, the application of PLA methacrylate, modified via the urethane formation reaction in a solution, in 3D laser stereolithography is hindered by partial crosslinking. This results in a decrease of the optical transparency of films prepared from the polymer and worsening of the conditions for photoprinting of 3D structures. At the same time, methacrylated PLA obtained in the supercritical carbon dioxide medium is optically transparent and does not limit the possibilities of laser 3D photoprinting. A typical structure fabricated by two-photon polymerization from polylactide methacrylated in the scCO_2_ medium is presented in Figure 11.

### 3.3. Study of the Local Stiffness of Crosslinked Structures

**Figure 12:**
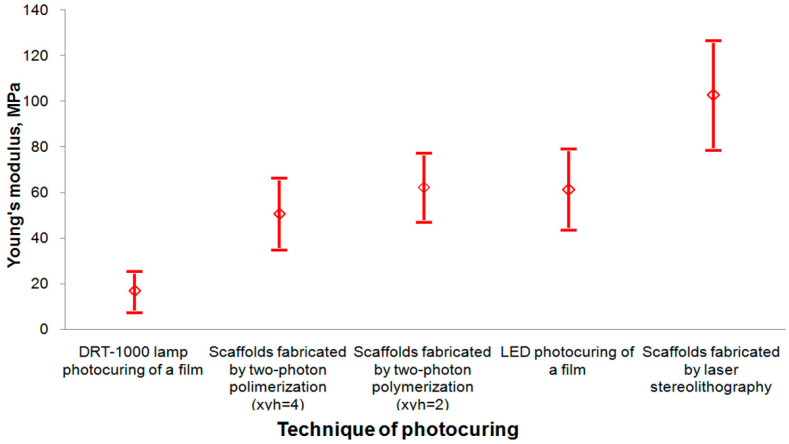
Dependence of Young’s modulus of 3D crosslinked structures (films and scaffolds) on the photocuring technique. DRT-1000 lamp photocuring of a film: E = 16.9 MPa (standard deviation (SD) is ±8.2 MPa). Scaffolds fabricated by two-photon polymerization (xyh = 4): E = 50.8 MPa (SD is ±15 MPa). Scaffolds fabricated by two-photon polymerization (xyh = 2): E = 62.2 MPa (SD is ±14.3 MPa). LED photocuring of a film: E = 61.3 MPa (SD is ±18.5 MPa, the relative error is 6%). Scaffolds fabricated by laser stereolithography: E = 102.5 MPa (SD is ±23.2 MPa). The composition of the mixture for photopolymerization is as follows: methacrylated PLA, 15 wt. % of OUDM and 5 wt. % of the PI (propidium iodide). The OUDM and PI contents are calculated with respect to the PLA weight.

The Young’s modulus (E) of scaffolds (measured by nanoindentation) appears to depend not only on the crosslinker content but also on the photocuring technique. In Figure 12, effective Young’s moduli are presented, which are obtained by averaging the corresponding local values for samples of polylactide films and scaffolds photocured by different techniques. The standard deviation for each curing technique is also displayed, and their values are presented in the figure legend. As seen from Figure 12, the highest Young’s modulus value is reached for scaffolds fabricated by laser stereolithography. The dispersion in the values for different samples is related to the surface inhomogeneity and depends on the technique of their preparation: from the highest dispersion for mercury lamp irradiation to the lowest dispersion for laser stereolithography, which corresponds to the increase in the crosslinking homogeneity. Besides, multilayered scaffolds of a certain shape are more conveniently prepared by two-photon polymerization. The process of two-photon absorption causing photopolymerization occurs in an extremely localized space resembling a prolate ellipsoid, which is located in the laser beam waist (in the experiments, the height is ~5 μm, the diameter in the XY plane is ~3 μm). This fact allows the formation of sophisticated structures of an optional design, setting their density by variation of the distance between individual passes of the laser beam—XY-hatch (xyh, nm).

### 3.4. DTA Study of Crosslinked Samples

**Figure 13:**
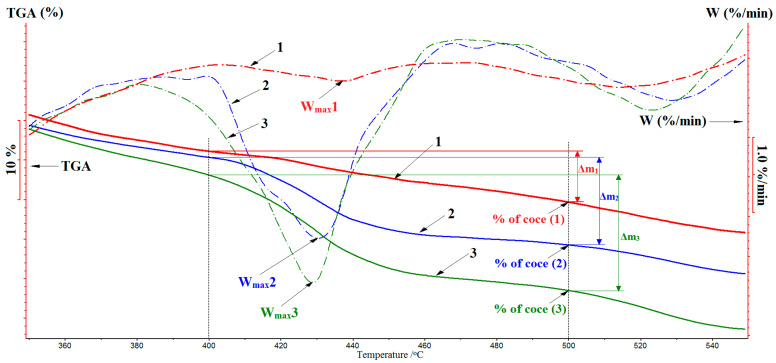
Thermooxidative destruction curves of photocured methacrylated PLA obtained by: 1—laser stereolithography, 2—LED irradiation, 3—mercury lamp irradiation. The weight loss (TGA, %) is shown by solid lines, and the rate of weight loss (W, %/min) is shown by dash-dotted lines. See the photocuring conditions in the Materials and Methods.

The degree of crosslinking of the obtained systems is also estimated based on the coke residue in the TGA analysis of crosslinked PLA methacrylates (Figure 13, Table 1). The value of the coke residue after the polymer destruction reflects the degree of the matrix crosslinking since crosslinked structural fragments are carbonized and form a coke residue at elevated temperatures. According to the DTA data (Figure 13), in the destruction of methacrylated PLA cured by laser stereolithography, the coke residue is 15%, while a LED-photocured sample forms only 9% of the coke. At the same time, the modified PLA irradiated by the mercury lamp is completely destructed, without the formation of a coke residue, which testifies the absence of crosslinked fragments in its structure. Thus, the highest degree of crosslinking is observed for samples cured by laser stereolithography.

The above conclusion is confirmed also by the character of the weight loss (TGA) in the thermooxidative destruction of samples undergone different photocuring procedures. Indeed, as seen from the TGA curves (Figure 13), the main weight loss (Δm) of samples occurs in the temperature range of 400–500 °C, the highest Δm being observed for samples irradiated with the mercury lamp, which are burned almost completely (curve 3). At the same time, Δm is decreased for samples that undergo the LED irradiation (curve 2), and this stage almost disappears in the thermooxidative destruction of samples obtained by laser stereolithography (curve 1) with the formation of the highest amount of the coke residue. This finding also indicates that structural fragments of the modified PLA are crosslinked to the highest degree in the conditions of laser stereolithography than they are during the other procedures. The decrease of the rate of destruction when going from mercury lamp-photocured samples to samples cured by laser stereolithography testifies this fact, as well (Table 1).

### 3.5. Cytotoxicity Analysis

**Figure 14:**
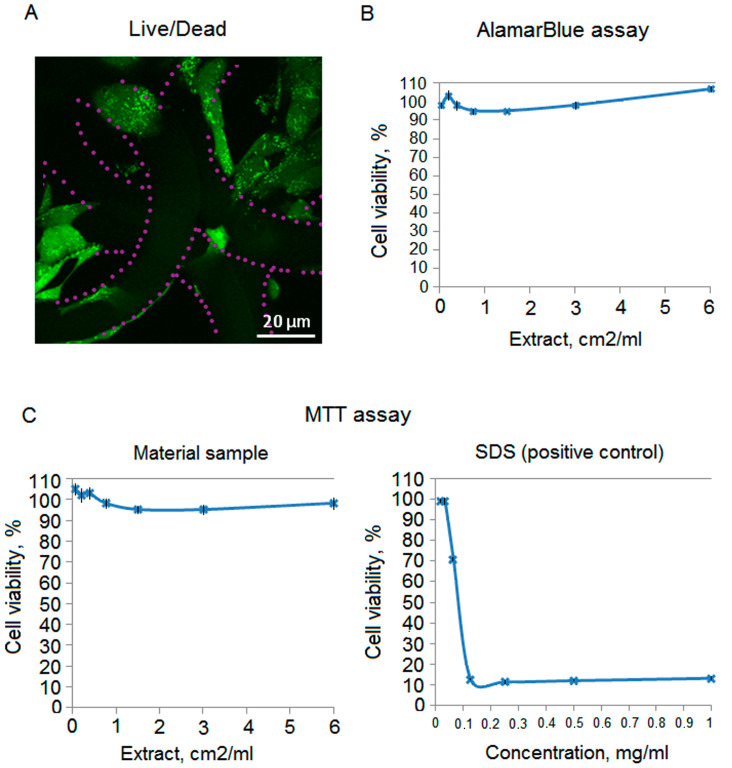
Cytotoxicity analysis of a crosslinked structure (scaffold) obtained by two-photon polymerization, consisting of the modified methacrylate-containing PLA, 15 wt. % of OUDM, and 5% of a photoinitiator: (**A**)—Live/dead staining (live cells—green (Calcein AM), dead cells—red (propidium iodide), violet dots—scaffold). Confocal microscopy; (**B**)—AlamarBlue assay; (**C**)—MTT assay. SDS is used as a positive control.

Scaffolds and films from the material containing 15 wt. % of OUDM are tested for cytotoxicity using live/dead staining and AlamarBlue and MTT-assays (Figure 14), which are standard for the preliminary assessment of new materials before in vivo experiments [46,47,52,53,54]. Live/dead staining is widely applied and based on two dyes—calcein-AM and ethidium homodimer-1 (propidium iodide), which help to visualize the cell state. While penetrating into a viable cell, the first dye indicates the intracellular esterase activity; however, the second one can stain only dead cells with the increased membrane permeability. Both MTT and AlamarBlue assays are based on metabolic reactions that occurred in mitochondria; however, their mechanisms are different. In the first case, tetrazolium salt is mainly transformed by mitochondrial succinic dehydrogenases of viable cells; so, water-insoluble formazan crystals form. In the second case, the reduction of AlamarBlue (resazurin) to resorufin occurs, which causes the medium color change and increase in fluorescence [55]. In our experiments, live/dead staining has shown that in 72 h after the inoculation, fibroblasts are attached to the matrix surface, spread on it, and have a typical morphology (bipolar or multipolar elongated cells); no dead or rounded cells are observed (Figure 14A). Using AlamarBlue and MTT assays, we revealed no significant cytotoxicity because the cell viability for all the dilutions of the matrix extract is higher than 70% (Figure 14B,C). SDS is used as a positive control to show that the used cell culture is susceptible to toxic agents. The revealed data correlates with the previously reported results on other PLA modifications. Particularly, tetrafunctional PLA structured using two-photon polymerization also shows no cytotoxic effects and induces bone formation in vivo [48].

## 4. Conclusions

We have realized a two-stage modification of polylactide in the supercritical carbon dioxide medium with the introduction of unsaturated methacrylate groups into PLA macromolecules. The optimal ratio of the initial components (based on the corresponding study of the modification efficiency) and the two-stage reaction design in the supercritical fluid medium have allowed obtaining a methacrylate PLA derivative with the degree of modification exceeding 80%. The modified polylactide has been shown suitable for photostructuring using such techniques as photocuring with UV irradiation of a mercury lamp or a LED, 3D printing by stereolithography, or two-photon polymerization. Using the most accurate and promising technique of two-photon polymerization, we have fabricated 3D crosslinked structures (scaffolds) from the modified PLA. The optimal content of the crosslinking agent (OUDM), providing the formation of stable stiff crosslinked structures, is found to be about 15%. The Young’s modulus of the prepared structures depends, among other factors, on the photocuring technique: the structures formed by laser stereolithography have the highest Young’s modulus. The DTA study has shown that Young’s moduli of the structures correlate with the degree of the polymer matrix crosslinking, which depends on the photocuring technique. As a study result, nontoxic and easy-to-handle material has been prepared, with the use of low toxic methacrylate modifying agents and an environmentally friendly solvent, scCO_2_. The material is capable of structuring with laser additive technologies for application in tissue engineering.

## Figures and Tables

**Table 1:** The thermooxidative destruction parameters of modified PLA, cured by different techniques.

No.	Technique of Photocuring *	Δm 400–500 °C, %	W_Max_, %/min	% of Coke Residue at 500 °C
1	Laser stereolithography (λ = 263 nm, 0.2 W/cm^2^)	6	0.7	15
2	LED photocuring of a film (λ = 365 nm, 5 W/cm^2^)	11	2.7	9
3	DRT-1000 lamp photocuring of a film (λ = 253 nm, ≈0.03 W/cm^2^)	15	4.4	0

* See the photocuring conditions on the Materials and Methods, where Δm is the weight loss in the temperature range of 400–500 °C, W_Max_ is the highest rate of the weight loss in the temperature range of 400–500 °C.

## Supplementary Materials

The following are available online at https://www.mdpi.com/2073-4360/12/11/2525/s1, Figure S1: The initial SEC elution profiles of the re-precipitation-purified product mixture, obtained at the second stage, Figure S2a. ^1^H-NMR spectrum of the initial polylactide (^1^H-NMR ((CDCl_3_) 600 MHz) δ, ppm: 1.48–1.66 (m, 3H, CH_3_), 4.37 (d, 1H, CH), 5.27–5.16 (m, 1H, CH)), Figure S2b. ^1^H-NMR spectrum of the initial 3-isocyanatomethyl-3,5,5-trimethylcyclohexyl isocyanate (^1^H-NMR ((CDCl_3_) 600 MHz) δ, ppm: 0.99, 1.01 (d, 9H, CH3_c,d_), 1.05, 1.08 (d, 6H, CH2_e_), 1.09–1.94 (15H, CH3_c,d_, CH2_e_), 3.07 (s, 2H, CH2_f_), 3.3 (q, 2H, CH_f_), 3.55, 3.67 (m, 1H, CH_h_)), Figure S2c. ^1^H-NMR spectrum of the initial ethyleneglycol monomethacrylate (^1^H-NMR ((CDCl_3_) 600 MHz) δ, ppm: 1.97 (s, 3H, CH3_n_), 2.17 (s, 1H, OH_p_), 3.87 (t, 2H, CH2_j_), 4.30 (t, 2H, CH2_k_), 5.61 (s, 1H, CH2_l,m_), 6.15 (s, 1H, CH2_l,m_)).
