# Supplementary material for: Photocurable Methacrylate Derivatives of Polylactide: A Two-Stage Synthesis in Supercritical Carbon Dioxide and 3D Laser Structuring
Source: Polymers (Basel). 2020 Oct 29;12(11):2525. doi: 10.3390/polym12112525 (PMC7692848; doi:10.3390/polym12112525)
Supplement: Supplementary file 1 [file polymers-12-02525-s001.pdf]

ÈÕÔ ĐÀÍ

Project Name: Defaults

Reported by User: System

SAMPLE INFORMATION

Sample Name:	Pla+OUM	Acquired By:	System
Sample Type:	Broad Unknown	Date Acquired:	24.01.2019 13:40:31
Vial:	1	Acq. Method:	base IR UV
Injection #:	1	Date Processed:	24.01.2019 15:10:19
Injection Volume:	20.00 ul	Channel Name:	410
Run Time:	34.00 Minutes	Sample Set Name:	

Autoscaled Chromatogram

20.00

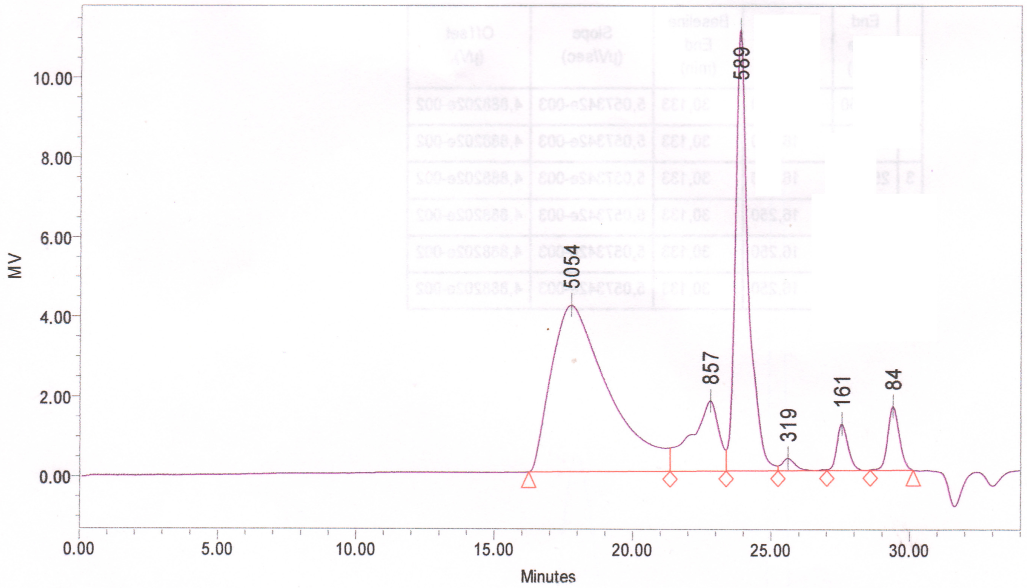

GPC Results

	Dist Name	Elution Volume (ml)	Retention Time (min)	Adjusted RT (min)	Mn	Mw	MP	Mz	Mz+1	Mz/Mw
1		17.757	17.757	17.757	3777	4367	5054	4868	5272	1.114730
2		22.800	22.800	22.800			857			
3		23.862	23.862	23.862			589			
4		25.606	25.606	25.606			319			
5		27.547	27.547	27.547			161			
6		29.392	29.392	29.392			84			

GPC Results

	Mz+1/Mw	Area (µV*sec)	% Area	Height (µV)	% Height	Integration Type	Peak Codes	Points Across Peak	Start Time (min)
1	1.207345	597705	51.05	4168	20.80	bv		305	16.250

Report Method: Áåçûìÿíûé Printed 16:05:36 24.01.2019 Page: 1 of 6

Figure S1. The initial SEC elution profiles of the re-precipitation-purified product mixture, obtained at the second stage.

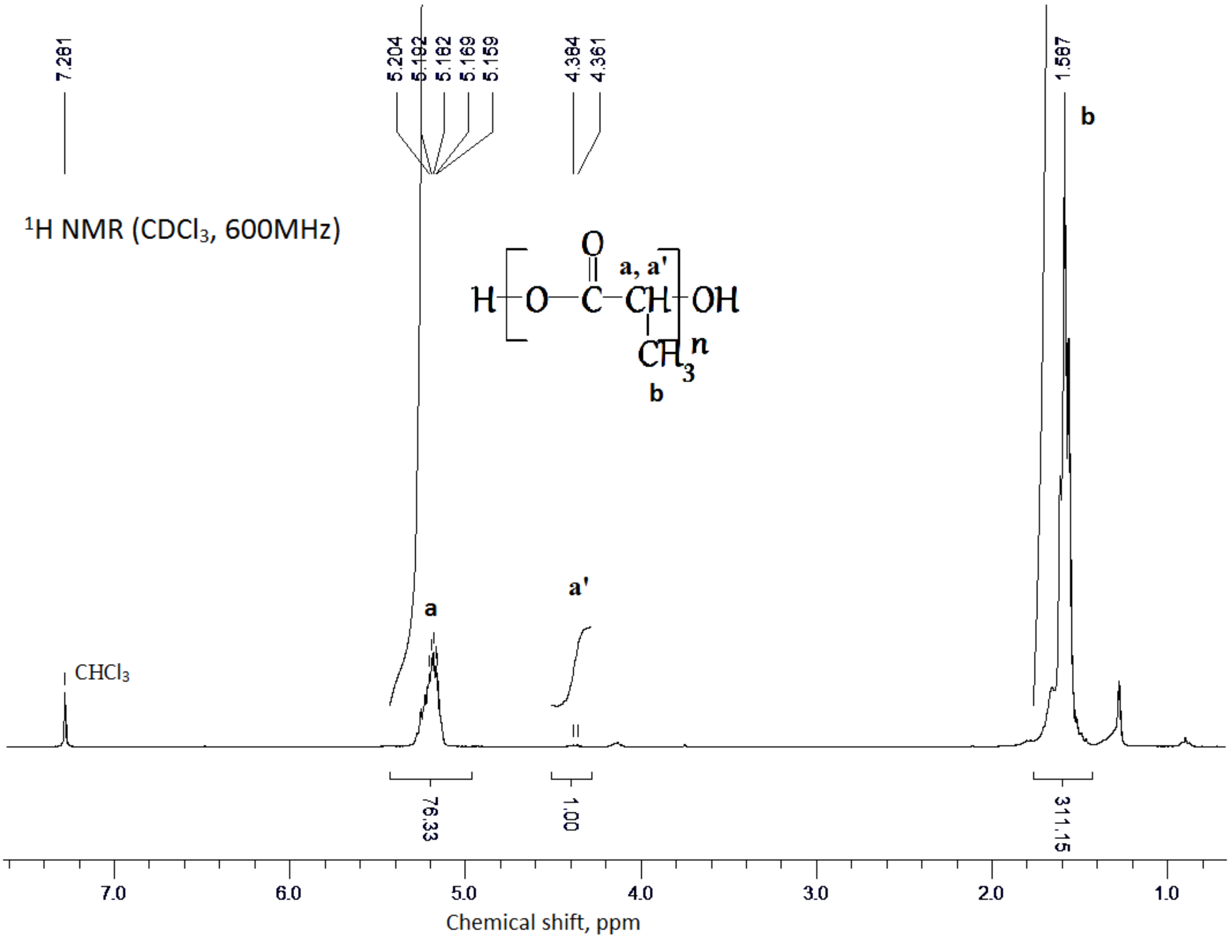

Figure S2a. ^{1}H-NMR spectrum of the initial polylactide.

^{1}H-NMR (($CDCl_3$) 600 MHz) δ, ppm: 1.48 – 1.66 (m, 3H, CH3), 4.37 (d, 1H, CH), 5.27 – 5.16 (m, 1H, CH).

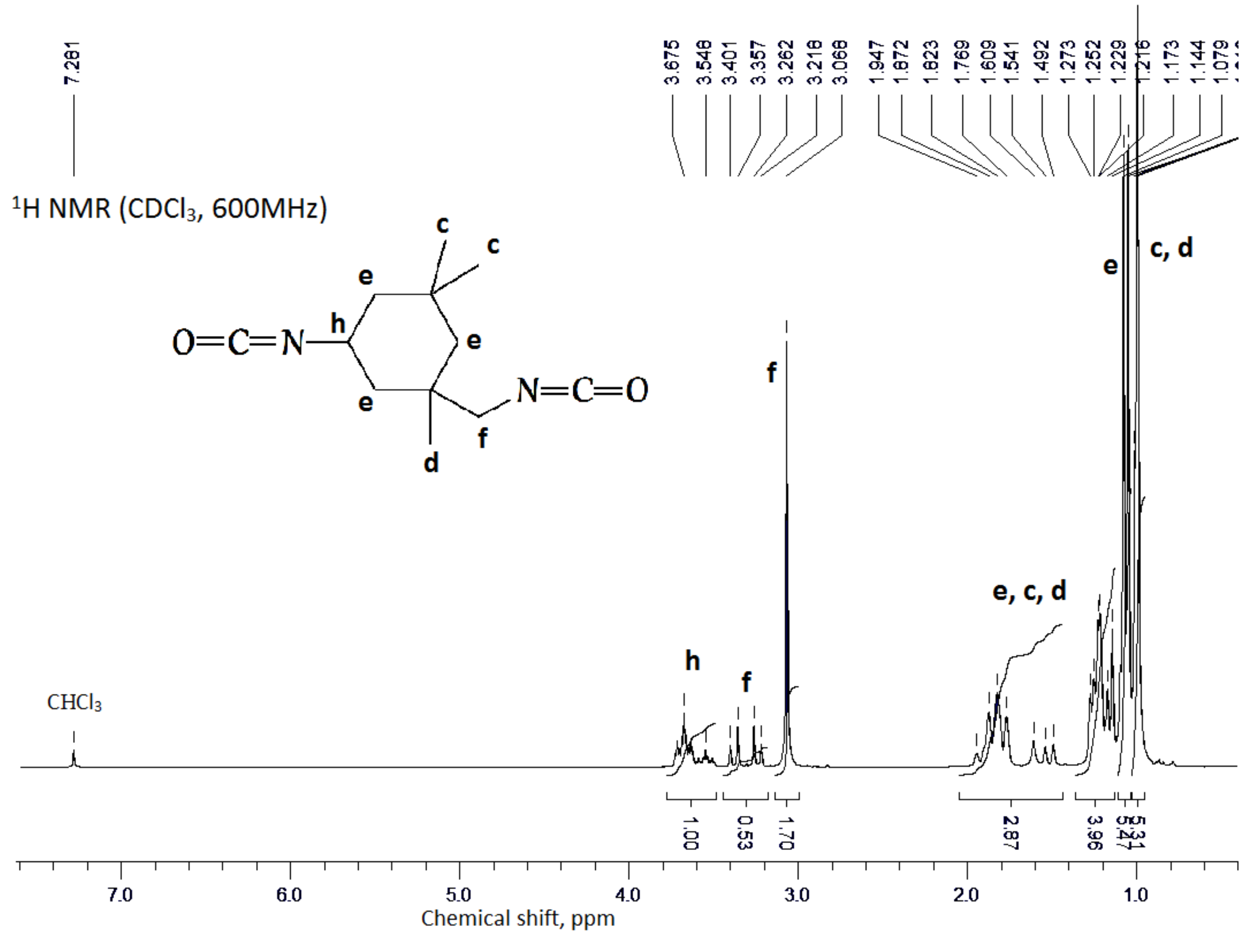

Figure S2b. ^{1}H-NMR spectrum of the initial 3-isocyanatomethyl-3,5,5-trimethylcyclohexyl isocyanate.

^{1}H-NMR (($CDCl_3$) 600 MHz) δ, ppm: 0.99, 1.01 (d, 9H, $CH3_{c,d}$), 1.05, 1.08 (d, 6H, $CH2_e$), 1.09 – 1.94 (15H, $CH3_{c,d}$, $CH2_e$), 3.07 (s, 2H, $CH2_f$), 3.3 (q, 2H, CH_f), 3.55, 3.67 (m, 1H, CH_h).

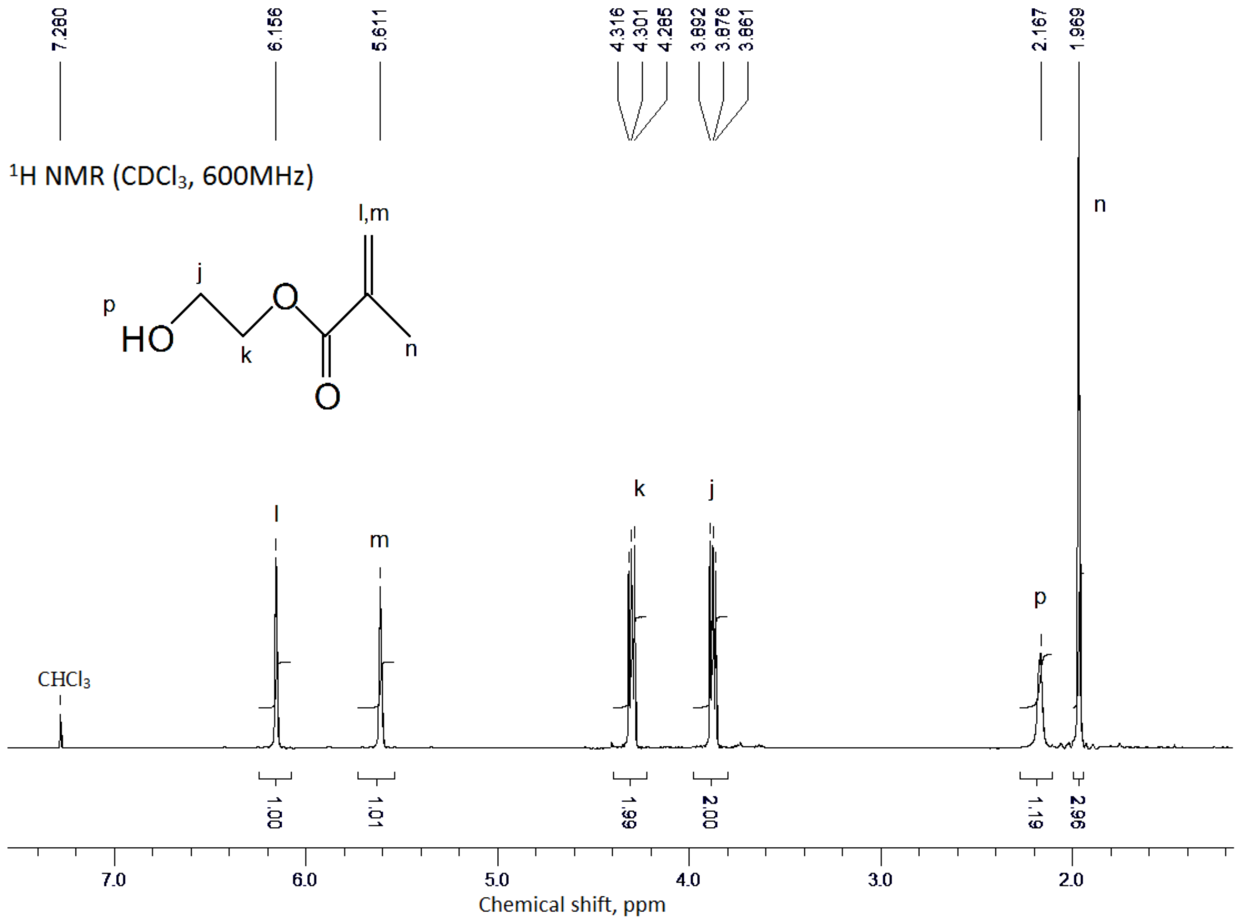

Figure S2c. ^{1}H-NMR spectrum of the initial ethyleneglycol monomethacrylate.
^{1}H-NMR (($CDCl_3$) 600 MHz) δ, ppm: 1.97 (s, 3H, $CH3_n$), 2.17 (s, 1H, OH_p), 3.87 (t, 2H, $CH2_j$), 4.30 (t, 2H, $CH2_k$), 5.61 (s, 1H, $CH2_{l,m}$), 6.15 (s, 1H, $CH2_{l,m}$).
